# Parameter Interval Estimation of System Reliability for Repairable Multistate Series-Parallel System with Fuzzy Data

**DOI:** 10.1155/2014/275374

**Published:** 2014-05-22

**Authors:** Wimonmas Bamrungsetthapong, Adisak Pongpullponsak

**Affiliations:** Department of Mathematics, Faculty of Science, King Mongkut's University of Technology Thonburi, Bangkok 10140, Thailand

## Abstract

The purpose of this paper is to create an interval estimation of the fuzzy system reliability for the repairable multistate series–parallel system (RMSS). Two-sided fuzzy confidence interval for the fuzzy system reliability is constructed. The performance of fuzzy confidence interval is considered based on the coverage probability and the expected length. In order to obtain the fuzzy system reliability, the fuzzy sets theory is applied to the system reliability problem when dealing with uncertainties in the RMSS. The fuzzy number with a triangular membership function is used for constructing the fuzzy failure rate and the fuzzy repair rate in the fuzzy reliability for the RMSS. The result shows that the good interval estimator for the fuzzy confidence interval is the obtained coverage probabilities the expected confidence coefficient with the narrowest expected length. The model presented herein is an effective estimation method when the sample size is *n* ≥ 100. In addition, the optimal ***α***-cut for the narrowest lower expected length and the narrowest upper expected length are considered.

## 1. Introduction 

Most researches on reliability theory involve traditional binary reliability models where each component in a system basically consists of two functional states, perfect functionality and complete failure. However, in the system reliability of multistate components, the entire system performance will be considered from different performance levels and several failure modes. The evolution of such a system is represented by a continuous-time discrete state stochastic process. The multistate system is widely used in various industrial areas such as power generation systems, computer systems, and transportation systems (Lisnianki and Levitin [1]). Compared with a binary system model, a multistate system (MSS) model provides a more flexible tool for representing engineering systems in real life as first introduced in [2–4]. Recent research has focused on reliability evaluation and optimization of MSS [5–7]. In conventional multistate theory, it is assumed that the exact probability of each component state is given. However, with the progress of modern industrial technologies, the product development cycles have become shorter, while the lifetime of products has become longer [8]. In many highly reliable applications, there may be only a few available observations of the system's failures. Therefore, it may be difficult to obtain sufficient data to estimate the precise values of the probabilities and performance levels of these systems. Moreover, the inaccuracy of system models, caused by human errors, is difficult to quantify using conventional reliability theory alone [9]. In light of these significant challenges, new techniques are needed to solve these fundamental problems related to reliability.

More recently, fuzzy reliability theory has been developed on the basis of fuzzy theory (Zadeh [10]). Presently, the applications of fuzzy idea in reliability theory that deal with the problem of lacking of inaccuracy or fluctuation data can be seen in many areas. In reliability analysis, many theories and methods have been constructed to facilitate the multistate system reliability assessment such as the universal generating function (Levitin [11]) and the multistate weight system (Li and Zuo [12]). Ding and Lisnianski [13] proposed the fuzzy universal generating function method to derive the fuzzy probability distribution and fuzzy system availability of the overall system when the component's performance rate and state probabilities take fuzzy values. Liu et al. [14] investigated the dynamic fuzzy system state probabilities, fuzzy availability, and fuzzy performance rewards of a multistate system under a continuous-time Markov model. Liu and Huang [15] proposed the fuzzy multistate system that has extended the multistate system model to the cases that the transition and performance rates of multistate elements are uncertain.

This research considers the problem of interval estimation of fuzzy system reliability when the parameter of interest is fuzzy and the data are observations from fuzzy random variables. A random sample of fuzzy data set is generated in constructing a fuzzy confidence interval. In some studies on fuzzy confidence interval, Corral and Gil [16] considered the problem of constructing confidence interval using fuzzy data without considering any fuzzy random variables. Geyer and Meeden [17] introduced a fuzzy confidence interval that is the optimality of UMP and UMPU test. Viertl [18] investigated statistical inference about an unknown parameter based on fuzzy observations and developed testing hypothesis for crisp parameter based on fuzzy data. Wu [19] proposed an approach based on fuzzy random variables for constructing a fuzzy confidence interval for an unknown fuzzy parameter. Škrjanc [20] introduced a method to define a fuzzy confidence interval that combines a fuzzy identification methodology with some idea from applied statistics in finding the confidence interval defined by the lower and upper fuzzy bounds. Chachi and Taheri [21] proposed a method to construct the one-sided and two-sided fuzzy confidence intervals for an unknown fuzzy parameter based on normal fuzzy random variable. Škrjanc [22] presented a new method of confidence interval identification for Takagi-Sugeno fuzzy models in the case of the data with regionally changeable variance. The method combines a fuzzy identification methodology with some ideas from applied statistics. Some studies showed that much research proposed an approach based on fuzzy data for constructing a fuzzy confidence interval, but without considering the performance analysis of interval estimator. Then the performance of fuzzy confidence interval will be assessed based on the coverage probability and the expected length in this research.

In this research, the fuzzy system reliability of RMSS is constructed, where the fuzzy failure rate and the fuzzy repair rate for each component are the triangular fuzzy number. An approach to construct interval estimation of the fuzzy system reliability of RMSS which subsequently will be used in estimation of the fuzzy confidence interval of fuzzy system reliability is developed. Finally, the analytic expression to find the coverage probability and the expected length that it is used to interpret the efficiency of fuzzy confidence interval are presented.

## 2. Materials and Methods

### 2.1. Markov Model for Multistate Element

A Markov model has been used for evaluating the expected number of failures at an arbitrary time interval in many practical cases and can be described as a Poisson process (Lisnianki and Levitin [1]).

**Definition 1 (see Ibe [23])**

A stochastic process {*X*(*t*) | *t* ≥ 0} is a continuous-time Markov chain if, for all *t*, Δ*t* ≥ 0 and nonnegative integers *i*, *j*, *k*,

(1) P[X(t+Δt)=j ∣ X(Δt)=i,X(u)=k,0≤u≤Δt]=P[X(t+Δt)=j ∣ X(Δt)=i].

This means that in a continuous-time Markov chain the conditional probability of the future state at time *t* + Δ*t* given the present state at *s* and all part state depends only on the present state and is independent of the past. If in addition *P*[*X*(*t* + Δ*t*) = *j* | *X*(Δ*t*) = *i*] is dependent on *s*, then the process {*X*(*t*) | *t* ≥ 0} is said to be time homogeneous or have the time homogeneity* property*. Time homogeneous Markov chains have stationary transition probabilities. Let *P*_*ij*_(*t*) = *P*[*X*(*t* + Δ*t*) = *j* | *X*(Δ*t*) = *i*] be the probability that a Markov chain in state *i* will be in state *j* after an additional time *t*. Thus, the *P*_*ij*_(*t*) is the transition probability function that satisfies the condition 0 ≤ *P*_*ij*_(*t*) ≤ 1. Also, ∑_*j*_ *P*_*ij*_(*t*) = 1.

### 2.2. Kolmogorov Differential Equations

Using a set of differential equations to find *P*_*ij*_(*t*) (Rausand and Hoyland [24]), it is start by considering the Chapman-Kolmogorov equations

(2) $$\begin{array}{c} P_{ij}\left(t+\Delta t\right)=\overset{r}{\underset{k=0}{\sum }}P_{ik}\left(\Delta t\right)P_{kj}\left(t\right). \end{array}$$

The interval of (0, *t* + Δ*t*) is split into two parts. First, consider a transition from state *i* to state *k* in the small interval (0  , Δ*t*) and then a transition from state *k* to state *j* in the rest of the interval. It is seen that

(3) $$\begin{array}{c} {\dot{P}}_{ij}\left(t\right)=\overset{r}{\underset{\begin{array}{c} k=0\\ k\neq i \end{array}}{\sum }}\alpha_{ik}P_{kj}\left(t\right)\;-\;\alpha_{i}P_{ij}\left(t\right)=\overset{r}{\underset{k=0}{\sum }}\alpha_{ik}P_{kj}\left(t\right), \end{array}$$

where *α*_*ii*_ = −*α*_*i*_ and the following notation for the time derivative ${\dot{P}}_{ij}(t)=(d/dx)P_{ij}(t)\;\;$is introduced. The differential equation (3) is known as the Kolmogorov backward equations. They are called backward equations because we start with a transition back by the start of the interval. The Kolmogorov backward equations may also be written in matrix format as

(4) $$\begin{array}{c} \dot{P}\left(t\right)=A\cdot P\left(t\right). \end{array}$$

Likewise, split the time interval (0, *t* + Δ*t*) into two parts. Consider a transition from *i* to *k* in the interval (0, *t*) and then a transition from *k* to *j* in the small interval (*t*, *t* + Δ*t*). It is seen that

(5) $$\begin{array}{c} {\dot{P}}_{ij}\left(t\right)=\overset{r}{\underset{\begin{array}{c} k=0\\ k\neq j \end{array}}{\sum }}\alpha_{kj}P_{ik}\left(t\right)\;-\;\alpha_{j}P_{ij}\left(t\right)=\overset{r}{\underset{k=0}{\sum }}\alpha_{kj}P_{ik}\left(t\right), \end{array}$$

where, as before, *α*_*jj*_ = −*α*_*j*_. The differential equation (5) is known as the Kolmogorov forward equations. The interchange of the limit and the sum above does not hold in all cases but is always valid when the state space is finite.

Consider the following:

(6) $$\begin{array}{c} \dot{P}\left(t\right)=P\left(t\right)\cdot A. \end{array}$$

For the Markov processes, the backward and the forward equations have the same unique solution  **P**(*t*), where ∑_*j*=0_^*r*^ *P*_*ij*_(*t*) = 1 for all *i* in *X*.

### 2.3. Repairable Multistate Elements

**Figure 1:**
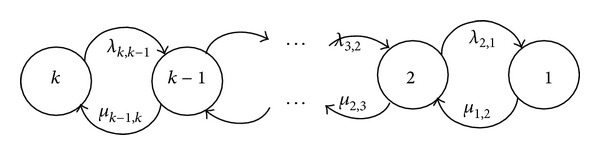
The state-space diagram of repairable multistate element with minor failures and minor repairs.

In this section, the repairable multistate element assumes that minor failures can happen and minor repairs can be done (Xie et al. [25]). A minor failure is a failure that causes the element transition from state *i* to state *i* − 1 denoted by *λ*_*i*,*i*−1_. On the other hand, a minor repair is a repair that causes the element transition from state *i* − 1 to state *i* denoted by *μ*_*i*−1,*i*_. It is actually a birth and death process as presented in Figure 1.

The Chapman-Kolmogorov equations for the general case are as follows:

(7) dP1(t)dt=−μ1,2P1(t)+λ2,1P2(t),dP2(t)dt=μ1,2P1(t)−(λ2,1+μ2,3)P2(t)+λ3,2P3(t),⋯=⋯,dPk(t)dt=μk−1,kPk−1(t)−λk,k−1Pk(t).

Assume that the initial state is in the state *k* with the best performance. Therefore, by solving (7) of differential equations under the initial condition *P*_*k*_(0) = 1, *P*_*k*−1_(0) =   *P*_*k*−2_(0) = ⋯ = *P*_1_(0)   =   0. The unreliability function for the multistate element will be a sum of the probabilities of the unacceptable states 1,…, *i*. Therefore reliability function is given by

(8) $$\begin{array}{c} R_{\text{repair}}\left(t\right)=1-\overset{i}{\underset{j=1}{\sum }}P_{j}\left(t\right). \end{array}$$

### 2.4. Basic Concept of Fuzzy Set Theory

The basic concepts which are used for analysing the fuzzy system reliability are discussed in this section. In classical set theory, an element *x* in a universe *U* may or may not be a membership of some crisp set *A*. This binary membership can be represented by the following indicator function:

(9) $$\begin{array}{c} \chi_{A}\left(x\right)=\{\begin{array}{cc} 1 & \text{if}\;\;x\in A\\ 0 & \text{if}\;\;x\notin A. \end{array} \end{array}$$

Zadeh's [10] extended the notation of binary membership to accommodate various degrees of membership on the real continuous interval [0,1] and defined the fuzzy set $\tilde{A}$ by the membership function $\xi_{\tilde{A}}(x)\in \left[0,1\right]$, given that $\xi_{\tilde{A}}(x)$ is the degree of membership of element *x* in fuzzy set $\tilde{A}=\left\{\left(x,\xi_{\tilde{A}}(x)\right);x\in X\right\}\;$. Consider a closed interval of real numbers [*a*, *b*] = {*x* ∈ *R* | *a* ≤ *x* ≤ *b*} ∀ *a*, *b* ∈ *R*. The following are formulas for four basic arithmetic operations on closed intervals of real numbers (Ross [26]):

(10) [a,b]+[c,d]=[a+c,b+d],[a,b]−[c,d]=[a−d,b−c],[a,b]·[c,d]=[min⁡⁡(ac,ad,bc,bd),max⁡⁡(ac,ad,bc,bd)],[a,b][c,d]=[a,b][1/c,1/d]    when    0∈[c,d].

The triangular membership function of fuzzy set $\tilde{A}$ is given by

(11) $$\begin{array}{c} \xi_{\tilde{A}}=\{\begin{array}{cc} 0; & x<L\\ \frac{x-L}{M-L}; & \;L\leq x\leq M\\ \frac{U-x}{U-M}; & M\leq x\leq U\\ 0; & x>U. \end{array} \end{array}$$

**Figure 2:**
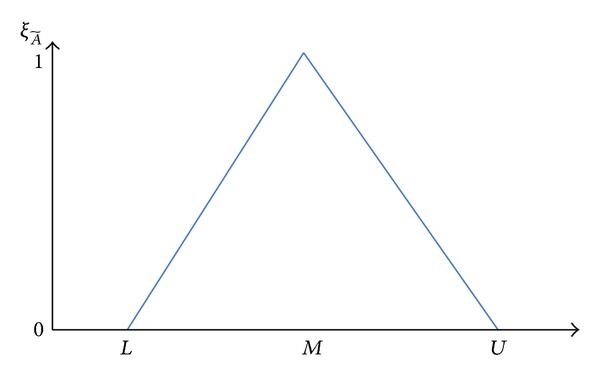
Fuzzy input membership function (the triangular membership function).

Let *R* be a universal set of real numbers and $\tilde{A}$ a fuzzy subset of *R*. $\tilde{A}$ is referred to as triangular fuzzy number (TFN) as shown in Figure 2, denoted by $\tilde{A}=(L,M,U)$. Let ${\tilde{A}}_{\alpha }$ be the *α*-cut level, so we have

(12) A~α=[A~αL,A~αU]=[L+α(M−L),U−α(U−M)];    ∀α∈[0,1].

### 2.5. Repairable Fuzzy Multistate Elements

Based on the conventional multistate elements, the state space diagram of a repairable multistate system takes the form presented in Figure 1 where state *k* is the best state and state 1 is the worst state. The minor failures between states *i* and *i* − 1 are determined by fuzzy value ${\tilde{\lambda }}_{i,i-1}$. And the minor repairs between states *i* − 1 and *i* are determined by fuzzy value ${\tilde{\mu }}_{i-1,i}$. With the fuzzy transition intensities, the state probability of elements at time *t* must also be a fuzzy value ${\tilde{P}}_{i}(t)$, and then the Chapman-Kolmogorov equation with fuzzy transition intensities can be written as

(13) dP~1(t)dt=−μ~1,2P~1(t)+λ~2,1P~2(t),dP~2(t)dt=μ~1,2P~1(t)−(λ~2,1+μ~2,3)P~2(t)+λ~3,2P~3(t),⋯=⋯dP~k(t)dt=μ~k−1,kP~k−1(t)−λ~k,k−1P~k(t),

where the initial conditions are ${\tilde{P}}_{k}(0)=1$ and ${\tilde{P}}_{k-1}\left(0\right)={\tilde{P}}_{k-2}\left(0\right)=\cdots ={\tilde{P}}_{1}(0)=0$. The unreliability function for the multistate element will be a sum of the probabilities of the unacceptable states 1,…, *i*  (∑_*j*=1_^*i*^ *P*_*j*_(*t*)). Laplace transform is adopted to transform (13) into linear equation as follows:

(14) sP~1(s)=−μ~1,2P~1(s)+λ~2,1P~2(s),sP~2(s)=μ~1,2P~1(s)−(λ~2,1+μ~2,3)P~2(s)  +λ~3,2P~3(s),⋯=⋯sP~k(s)−1=μ~k−1,kP~k−1(s)−λ~k,k−1P~k(s),

where $s{\tilde{P}}_{i}(s)-{\tilde{P}}_{i}(0)=L\left[d{\tilde{P}}_{i}(t)/dt\right]$. Given that ${\tilde{P}}_{i}(s)$ is a function of $\tilde{\lambda }$, $\tilde{\mu }$, and *s*, then the inverse Laplace transform is executed to get the ${\tilde{P}}_{i}(t)$ in time domain:

(15) $$\begin{array}{c} {\tilde{P}}_{i}\left(t\right)=L^{-1}\left[{\tilde{P}}_{i}\left(s\right)\right]={{\tilde{P}}_{i}}^{*}\left(\tilde{\lambda },t\right), \end{array}$$

where *L* ^−1^[·] is an inverse Laplace operator and ${\tilde{P}}_{i}(t)$ is a function in terms of fuzzy variables $\tilde{\lambda }=\left\{{\tilde{\lambda }}_{k,k-1},\ldots ,{\tilde{\lambda }}_{i,j},\ldots ,{\tilde{\lambda }}_{2,1}\right\}$ and $\tilde{\mu }=\left\{{\tilde{\mu }}_{k-1,k},\ldots ,{\tilde{\mu }}_{j,i},\ldots ,{\tilde{\mu }}_{1,2}\right\}$ at any time *t*. The fuzzy state probabilities can be obtained in the form of ${\tilde{P}}_{i}(t)$ at the *α*-cut level:

(16) $$\begin{array}{c} {\tilde{P}}_{i\alpha }\left(t\right)\;=\left[{\tilde{P}}_{i\alpha }^{L}\left(t\right),{\tilde{P}}_{i\alpha }^{U}\left(t\right)\right];\;t\geq 0,\;\;0\leq \alpha \leq 1, \end{array}$$

where ${{\tilde{P}}^{L}}_{i\alpha }(t)=\min{{\tilde{P}}_{i}}^{*}\left(\lambda ,t;\mu_{\lambda_{i,j}}\left(\lambda_{i,j}\right)\geq \alpha \right)$ and ${\tilde{P}}_{i\alpha }^{U}\left(t\right)=\max{\tilde{P}}_{i}^{*}(\lambda ,t;\mu_{\lambda_{i,j}}(\lambda_{i,j})\geq \alpha )$.

Then the fuzzy reliability of repairable multistate element is given by

(17) $$\begin{array}{c} \left[{\tilde{R}}_{\alpha }^{L}\left(t\right),{\tilde{R}}_{\alpha }^{U}\left(t\right)\right]=\left[1-\overset{n}{\underset{i=1}{\sum }}{\tilde{P}}_{i\alpha }^{U}\left(t\right),1-\overset{n}{\underset{i=1}{\sum }}{\tilde{P}}_{i\alpha }^{L}\left(t\right)\right]. \end{array}$$

The fuzzy state probabilities can be obtained in the form that ${\tilde{P}}_{i\alpha }(t)=\;[{\tilde{P}}_{i\alpha }^{L}(t),{\tilde{P}}_{i\alpha }^{U}(t)]$ at the *α*-cut level where *α* ∈ [0,1]. Let $\;{\tilde{F}}_{i\alpha }^{}(t)$ be fuzzy unreliability functions of repairable multistate element in each element $\left(\;{\tilde{F}}_{i\alpha }^{}(t)=1-{\tilde{R}}_{i\alpha }^{}(t)\right)$.

### 2.6. Multistate Element under Series-Parallel System

**Figure 3:**
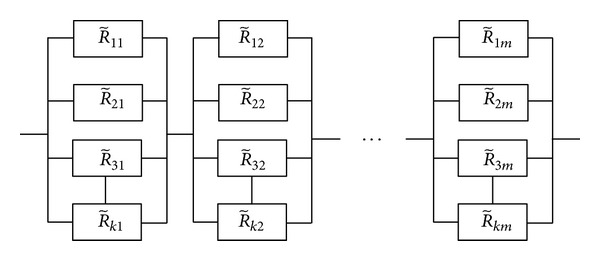
Fuzzy series-parallel system.

In this section, analysis of the fuzzy system reliability using the repairable multistate element with series-parallel system will be demonstrated.  Figure 3 represents a system containing *m* subsystem connection in series where each subsystem consists of *k* components in parallel. Let ${\tilde{R}}_{}^{ij}$ be a fuzzy reliability function and ${\tilde{F}}_{}^{ij}$ a fuzzy unreliability function of subsystem *i*, which is connected in series, and component *j*, which is connected in parallel (*i* = 1,2,…, *m* and *j* = 1,2,…, *k*), respectively. Let a failure rate and a repair rate be represented by ${\tilde{\lambda }}_{}^{ij}$ and ${\tilde{\mu }}_{}^{ij}$ at time *t*, respectively.

Then the fuzzy system reliability of repairable multistate series-parallel system (RMSS) at time *t* is given by

(18) R~(sp)α=∏i=1m[(1−∏j=1k(1−(R~ij)αL)),(1−∏j=1k(1−(R~ij)αU))],α∈[0,1].

### 2.7. Fuzzy Confidence Interval Probability for Fuzzy Parameter

**Figure 4:**
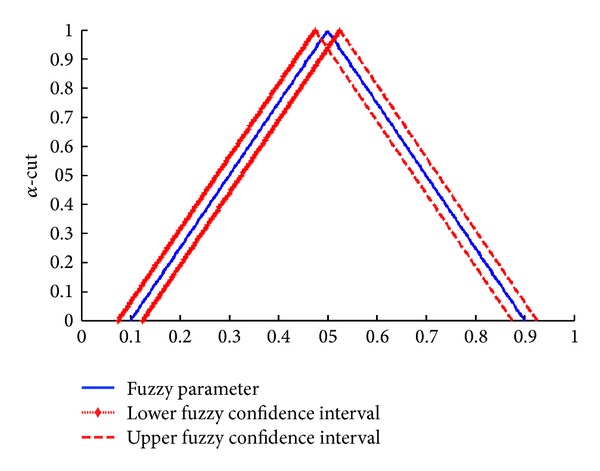
Two-sized 100(1 − *γ*)% confidence intervals for fuzzy parameter.

The interval estimation of the fuzzy system reliability of RMSS is constructed by extending the concepts of two-sided 100(1 − *γ*)% confidence interval of the real parameters to the case where both parameter and random variables are fuzzy as shown in Figure 4.

According to Wu [19], *R*_1_,…, *R*_*n*_ are independent and identically distributed random variables. Let *L*(**R**) and *U*(**R**) be two statistics such that *L*(**R**) ≤ *U*(**R**), where **R** = (*R*_1_,…, *R*_*n*_). If the random interval [*L*(**R**), *U*(**R**)] satisfies *P*[*L*(**R**) ≤ *θ* ≤ *U*(**R**)] = 1 − *γ*, then [*L*(**r**), *U*(**r**)] is a confidence interval for *θ* with confidence coefficient 1 − *γ*, where **r** = (*r*_1_,…, *r*_*n*_) and each *r*_*i*_ is an observed value of *R*_*i*_ when *i* = 1,2,…, *n*. It can be applied to fuzzy confidence interval. Let ${\tilde{R}}_{1},\ldots ,{\tilde{R}}_{n}$ be independent and identically distributed fuzzy random variables with fuzzy parameter $\tilde{\theta }$. Let ${\tilde{r}}_{i}$ be the observed value of ${\tilde{R}}_{i}$ for *i* = 1,2,…, *n*, where each ${\tilde{r}}_{i}$ is a fuzzy number for *i* = 1,2,…, *n*. Therefore, ${\tilde{r}}_{i\alpha }^{L}$ and ${\tilde{r}}_{i\alpha }^{U}$ are the observed values of ${\tilde{R}}_{i\alpha }^{L}$ and ${\tilde{R}}_{i\alpha }^{U}$ for *α* ∈ [0,1]. Then ${\tilde{R}}_{1\alpha }^{L},{\tilde{R}}_{2\alpha }^{L},\ldots ,{\tilde{R}}_{n\alpha }^{L}$ and ${\tilde{R}}_{1\alpha }^{U},{\tilde{R}}_{2\alpha }^{U},\ldots ,{\tilde{R}}_{n\alpha }^{U}$ are identically distributed fuzzy random variables. Suppose that the distribution of ${\tilde{R}}_{i}$ is unknown for *i* = 1,2,…, *n*. Then the approximate fuzzy confidence interval can be constructed using the central limit theorem when the sample size *n* is sufficiently large (Ross [27]). Let ${\tilde{R}}_{1},\ldots ,{\tilde{R}}_{n}$ be independent and identically distributed fuzzy random variables. There exists a unique fuzzy number $E\left(\tilde{R}\right)=\left(E\left({\tilde{R}}_{\alpha }^{L}\right),E\left({\tilde{R}}_{\alpha }^{U}\right)\right)$ where $\tilde{R}=\left({\tilde{R}}_{\alpha }^{L},{\tilde{R}}_{\alpha }^{U}\right)$ for all *α* ∈ [0,1]; then $E\left(\tilde{R}\right)$ is called the expectation of $\tilde{R}$. Suppose that $E\left(\tilde{R}\right)={\tilde{\mu }}_{R}$, $E\left({\tilde{R}}_{\alpha }^{L}\right)={\left({\tilde{\mu }}_{R}\right)}_{\alpha }^{L}$, and $E\left({\tilde{R}}_{\alpha }^{U}\right)={\left({\tilde{\mu }}_{R}\right)}_{\alpha }^{U}$, respectively. Therefore ${\tilde{R}}_{i\alpha }^{L}$ and ${\tilde{R}}_{i\alpha }^{U}$ have finite expectations ${\left({\tilde{\mu }}_{R}\right)}_{\alpha }^{L}$ and ${\left({\tilde{\mu }}_{R}\right)}_{\alpha }^{U}$ with unknown variances for *i* = 1,2,…, *n* and *α* ∈ [0,1].

Let $\bar{{\tilde{R}}_{\alpha }^{L}}=\left(1/n\right)\sum_{i=1}^{n}{\tilde{R}}_{i\alpha }^{L}\text{and}\;\;\;\bar{{\tilde{R}}_{\alpha }^{U}}=(1/n)\sum_{i=1}^{n}{\tilde{R}}_{i\alpha }^{U}$ and let $S_{\alpha L}^{2}=\left(1/n\right)\sum_{i=1}^{n}{\left({\tilde{R}}_{i\alpha }^{L}-\bar{{\tilde{R}}_{\alpha }^{L}}\right)}^{2}$an $S_{\alpha U}^{2}=(1/n)\sum_{i=1}^{n}{\left({\tilde{R}}_{i\alpha }^{U}-\bar{{\tilde{R}}_{\alpha }^{U}}\right)}^{2}$.

It is shown that $S_{\alpha L}^{2}\to \mathrm{Var}\left({\tilde{R}}_{i\alpha }^{L}\right)$ and $S_{\alpha U}^{2}\to \mathrm{Var}\left({\tilde{R}}_{i\alpha }^{U}\right)$ as →*∞*. The central limit theorem gives that $\sqrt{n}\left(\bar{{\tilde{R}}_{\alpha }^{L}}-{\left({\tilde{\mu }}_{R}\right)}_{\alpha }^{L}\right)/S_{\alpha L}^{}$ as well as $\sqrt{n}\left(\bar{{\tilde{R}}_{\alpha }^{U}}-{\left({\tilde{\mu }}_{R}\right)}_{\alpha }^{U}\right)/S_{\alpha U}^{}$ has approximately *N*(0,1) distribution. Therefore the approximate 100(1 − *γ*)% fuzzy confidence interval for ${\left({\tilde{\mu }}_{R}\right)}_{\alpha }^{L}$ and ${\left({\tilde{\mu }}_{R}\right)}_{\alpha }^{U}$ is given by

(19) $$\begin{array}{c} \left[\overset{-}{{\tilde{R}}_{\alpha }^{L}}-z_{\gamma /2}\frac{S_{\alpha L}}{\sqrt{n}},\overset{-}{{\tilde{R}}_{\alpha }^{L}}+z_{\gamma /2}\frac{S_{\alpha L}}{\sqrt{n}}\right]\equiv \left[L\left({\tilde{R}}_{\alpha }^{L}\right),U\left({\tilde{R}}_{\alpha }^{L}\right)\right], \end{array}$$

(20) $$\begin{array}{c} \left[\overset{-}{{\tilde{R}}_{\alpha }^{U}}-z_{\gamma /2}\frac{S_{\alpha U}}{\sqrt{n}},\overset{-}{{\tilde{R}}_{\alpha }^{U}}+z_{\gamma /2}\frac{S_{\alpha U}}{\sqrt{n}}\right]\equiv \left[L\left({\tilde{R}}_{\alpha }^{U}\right),U\left({\tilde{R}}_{\alpha }^{U}\right)\right], \end{array}$$

respectively.

We assign $\left[L\left({\tilde{R}}_{\alpha }^{L}\right),U\left({\tilde{R}}_{\alpha }^{L}\right)\right]$ as lower fuzzy confidence interval and $\left[L\left({\tilde{R}}_{\alpha }^{U}\right),U\left({\tilde{R}}_{\alpha }^{U}\right)\right]$ as upper fuzzy confidence interval.

### 2.8. Coverage Probability and Expected Length of Fuzzy Confidence Interval

For an interval estimator [*L*(**R**), *U*(**R**)] of a true parameter *θ*, the coverage probability of [*L*(**R**), *U*(**R**)] is the probability that the random interval [*L*(**R**), *U*(**R**)] covers *θ*. It is denoted by *P*(*θ* ∈ [*L*(**R**), *U*(**R**)]). In this research, the performance of fuzzy confidence interval is assessed based on the coverage probability and the expected length. The analytic expressions for the coverage probability of fuzzy confidence interval of true parameter ${\tilde{\mu }}_{R\alpha }=\left[{\left({\tilde{\mu }}_{R}\right)}_{\alpha }^{L},{\left({\tilde{\mu }}_{R}\right)}_{\alpha }^{U}\right]$ are derived.

Let $P\left({\left({\tilde{\mu }}_{R}\right)}_{\alpha }^{L}\in \left[L\left({\tilde{R}}_{\alpha }^{L}\right),U\left({\tilde{R}}_{\alpha }^{L}\right)\right]\right)$ and $P\left({\left({\tilde{\mu }}_{R}\right)}_{\alpha }^{U}\in \left[L\left({\tilde{R}}_{\alpha }^{U}\right),U\left({\tilde{R}}_{\alpha }^{U}\right)\right]\right)$ be the coverage probability of fuzzy confidence interval $\left[L\left({\tilde{R}}_{\alpha }^{L}\right),U\left({\tilde{R}}_{\alpha }^{L}\right)\right]$ and $\left[L\left({\tilde{R}}_{\alpha }^{U}\right),U\left({\tilde{R}}_{\alpha }^{U}\right)\right]$, respectively. Then the lower coverage probability of fuzzy confidence interval of ${\left({\tilde{\mu }}_{R}\right)}_{\alpha }^{L}$ is given by

(21) P((μ~R)αL∈[L(R~αL),U(R~αL)])=P[L(R~αL)<(μ~R)αL<U(R~αL)]=P[A<Z<B]=E[I{A<Z<B}(τ)],

where

(22) $$\begin{array}{c} I_{\left\{A<Z<B\right\}}\left(\tau \right)=\{\begin{array}{cc} 1, & \text{if}\;\tau \in \left\{A<Z<B\right\}\\ 0, & \text{otherwise}\;, \end{array} \end{array}$$

where $A=\bar{{\tilde{R}}_{\alpha }^{L}}-U\left({\tilde{R}}_{\alpha }^{L}\right)/\left(S_{\alpha L}^{}/\sqrt{n}\right)$, $B=\bar{{\tilde{R}}_{\alpha }^{L}}-L\left({\tilde{R}}_{\alpha }^{L}\right)/\left(S_{\alpha L}^{}/\sqrt{n}\right)$, and *Z* is the standard normal distribution.

Likewise, the upper coverage probability of fuzzy confidence interval of ${\left({\tilde{\mu }}_{R}\right)}_{\alpha }^{U}$ is given by

(23) P((μ~R)αU∈[L(R~αU),U(R~αU)]) =P[L(R~αU)<(μ~R)αU<U(R~αU)] =P[C<z<D] =E[I{C<Z<D}(τ)],

where

(24) $$\begin{array}{c} I_{\left\{C<Z<D\right\}}\left(\tau \right)=\{\begin{array}{cc} 1, & \text{if}\;\tau \in \left\{C<Z<D\right\}\\ 0, & \text{otherwise}, \end{array} \end{array}$$

where $C=\bar{{\tilde{R}}_{\alpha }^{U}}-U\left({\tilde{R}}_{\alpha }^{U}\right)/\left(S_{\alpha U}^{}/\sqrt{n}\right)$, $D=\bar{{\tilde{R}}_{\alpha }^{U}}-L\left({\tilde{R}}_{\alpha }^{U}\right)/\left(S_{\alpha U}^{}/\sqrt{n}\right)$, and *Z* is the standard normal distribution.

### 2.9. General Procedure for Investigating the Coverage Probabilities and Expected Length for Fuzzy Confidence Interval of Fuzzy System Reliability

In this section, the coverage probability and expected length for fuzzy system reliability of RMSS is investigated. Suppose that fuzzy parameter (population mean) is denoted by ${\tilde{\mu }}_{R\alpha }=\left[{\left({\tilde{\mu }}_{R}\right)}_{\alpha }^{L},{\left({\tilde{\mu }}_{R}\right)}_{\alpha }^{U}\right]$. A method to calculate the fuzzy coverage probability and expected length for ${\tilde{\mu }}_{R\alpha }$ is demonstrated as follows.

**Step 1**

Generate fuzzy random variables from normal distribution with ${\tilde{R}}_{1\alpha }^{L},{\tilde{R}}_{2\alpha }^{L},\ldots ,{\tilde{R}}_{n\alpha }^{L}\sim N\left({\left({\tilde{\mu }}_{R}\right)}_{\alpha }^{L},\sigma^{2}\right)$ and ${\tilde{R}}_{1\alpha }^{U},{\tilde{R}}_{2\alpha }^{U},\ldots ,{\tilde{R}}_{n\alpha }^{U}\sim N\left({\left({\tilde{\mu }}_{R}\right)}_{\alpha }^{U},\sigma^{2}\right)$. The sample sizes of this example are *n* = 30,50,100,150,200 and *σ* ^2^ = 1 for *α* ∈ [0,1].

**Step 2**

Compute fuzzy sample mean $\left[\bar{{\tilde{R}}_{\alpha }^{L}},\bar{{\tilde{R}}_{\alpha }^{U}}\right]$ and fuzzy sample variance [*S*_*αL*_^2^, *S*_*αU*_^2^] for *α* ∈ [0,1].

**Step 3**

Compute fuzzy confidence intervals of ${\tilde{\mu }}_{R}=\left[{\left({\tilde{\mu }}_{R}\right)}_{\alpha }^{L},{\left({\tilde{\mu }}_{R}\right)}_{\alpha }^{U}\right]$ for *α* ∈ [0,1], the lower fuzzy confidence interval $\left[L\left({\tilde{R}}_{\alpha }^{L}\right),U\left({\tilde{R}}_{\alpha }^{L}\right)\right]$, and the upper fuzzy confidence interval $\left[L\left({\tilde{R}}_{\alpha }^{U}\right),U\left({\tilde{R}}_{\alpha }^{U}\right)\right]$.

**Step 4**

Compute the fuzzy coverage probability (*CP*_*α*_) and the expected length (*EL*_*α*_) for *α* ∈ [0,1],

(25) CPα=(Number  of  times  that  the  fuzzy  confidenceinterval  covers  the  true  parameter  in  each  α-cut) ×m−1,ELα=∑i=1mUi−Lim,

where *U*_*i*_ is the lower fuzzy confidence interval and *L*_*i*_ is the upper fuzzy confidence interval at repeated time *i*, *i* = 1,2,…, *m*.

**Step 5**

Repeat Steps 2–4 for a given condition.

After receiving the coverage probabilities and the expected length of the fuzzy confidence interval, the next step is to consider the sample size (*n*) that gives the coverage probability higher than the expected confidence coefficient, where the number of repeatition is *m* = 10,000. The *Z*-test statistic hypothesis testing is used in confirming the level of the coverage probability as follows:

*H*_0_: the coverage probability is not less than the expected confidence coefficient (*CP* ≥ *CP*_0_),
*H*_1_: the coverage probability is lower than the expected confidence coefficient (*CP* < *CP*_0_),

when the significance level is *γ* = 0.95 and the test statistic

(26) $$\begin{array}{c} Z=\frac{\hat{C}P-CP_{0}}{\sqrt{CP_{0}\left(1-CP_{0}\right)/m}}. \end{array}$$

Since the criterion in the test is *Z* < −*Z*_*γ*_, the hypothesis *H*_0_ cannot be rejected if

(27) $$\begin{array}{c} \hat{C}P\geq CP_{0}-Z_{\gamma }\sqrt{\frac{CP_{0}\left(1-CP_{0}\right)}{m}}, \end{array}$$

where *CP* is the coverage probability, $\hat{C}P$ is the coverage probability estimated from this study, *CP*_0_ is the expected confidence coefficient, and *m* is the repeated time.

## 3. Numerical Example and Results

**Figure 5:**
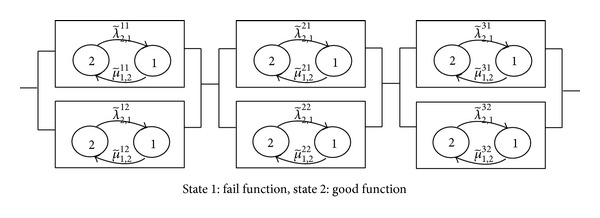
Multistate system and state diagrams for RMSS.

In this section, a RMSS which consists of 3 subsystems in series and 2 components in each subsystem in parallel is considered. In each element, it has repairable multistate with a fuzzy failure rate and a fuzzy repair rate as shown in Figure 5. Since a failure rate and a repair rate cannot be recorded precisely due to human errors, machine errors, or some unexpected situations, triangular fuzzy numbers are used to describe the fuzzy failure rate and fuzzy repair rate. The parameters of these functions are shown in Table 1.

For Markov model, the Chapman-Kolmogorov equations of each element can be written as

(28) dP~1ij(t)dt=−μ~1,2ijP~1ij(t)+λ~2,1ijP~2ij(t)dP~2ij(t)dt=μ~1,2ijP~1ij(t)−λ~2,1ijP~2ij(t). for  i=1,2,3, j=1,2  ,

where ${\tilde{P}}_{1}^{ij}(t)$ is probabilities of good function and ${\tilde{P}}_{2}^{ij}(t)$ is probabilities of fail function with fuzzy failure rate $\left({{\tilde{\lambda }}^{ij}}_{}\right)$ and fuzzy repair rate $\left({{\tilde{\mu }}^{ij}}_{}\right)$ for each element in multistate model. Using the inverse Laplace transform, the fuzzy state probabilities are obtained as functions of time *t* in the form of

(29) P~1ij(t)=  λ~ij2,1λ~ij2,1+μ~ij1,2(e−(λ~ij2,1+μ~ij1,2)·t−1)P~2ij(t)=    μ~ij1,2λ~ij2,1+μ~ij1,2+λ~ij2,1λ~ij2,1+μ~ij1,2e−(λ~ij2,1+μ~ij1,2)·tfor  i=1,2,3, j=1,2.

From Figure 5, fuzzy reliability functions and fuzzy unreliability functions of repairable for each element are given by

(30) R~ij(t)=P~2ij(t),F~ij(t)=P~1ij(t)for  i=1,2,3, j=1,2,

where ${\tilde{F}}_{}^{ij}(t)$ is fuzzy unreliability function of repairable multistate system at subsystem *i* connected in series and component *j* connected in parallel, respectively. Suppose that ${\tilde{\lambda }}_{2,1}^{ij}$ and ${\tilde{\mu }}_{2,1}^{ij}$are triangular membership function in each *α* ∈ [0,1], as shown in Table 1. It can be written in the form of *α*-cut as follows:

(31) [(λ~2,1ij)αL  ,(λ~2,1ij)αU]=[aij+α(bij−aij),cij−α(cij−bij)],[(μ~2,1ij)αL  ,(μ~2,1ij)αU]=[eij+α(eij−dij),fij−α(fij−eij)].

**Figure 6:**
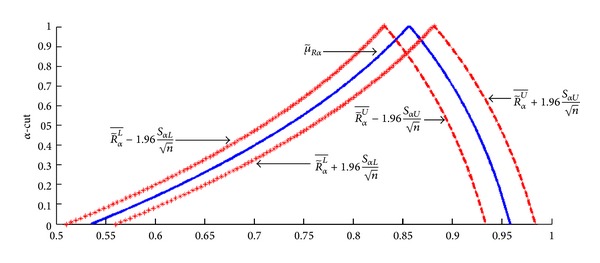
Fuzzy 95% confidence intervals for fuzzy parameter ${\tilde{\mu }}_{R\alpha }=\left[{\left({\tilde{\mu }}_{R}\right)}_{\alpha }^{L},{\left({\tilde{\mu }}_{R}\right)}_{\alpha }^{U}\right]$.

From the example system in Figure 6, RMSS with fuzzy failure rate ${\left({\tilde{\lambda }}_{2,1}^{ij}\right)}_{\alpha }$ and a fuzzy repair rate ${\left({\tilde{\mu }}_{2,1}^{ij}\right)}_{\alpha }$are considered. The fuzzy system reliability in each *α* ∈ [0,1] is given by

(32) R~sα=[R~sαL(t),R~sαU(t)]=∏i=13[[1,1]α−[∏j=12(1−(R~ij)αU),∏j=12(1−R~ij)αL]].

Substituting fuzzy reliability function $\left({\tilde{R}}_{}^{ij}(t)\right)$ in each element into (32), then the fuzzy system reliability of RMSS is shown in Table 2.

Suppose that fuzzy system reliability for RMSS in Table 2 is the fuzzy parameter (population mean) denoted by ${\tilde{\mu }}_{R\alpha }=\left[{\left({\tilde{\mu }}_{R}\right)}_{\alpha }^{L},{\left({\tilde{\mu }}_{R}\right)}_{\alpha }^{U}\right]$. The confidence coefficient with *γ* = 0.95 is defined and fuzzy random variables from normal distribution are generated by using MATLAB [28]. Then the fuzzy coverage probability and the expected length for ${\tilde{\mu }}_{R\alpha }$ are shown in Figure 6.

After receiving the coverage probabilities and the expected length of fuzzy confidence interval, the next step is to compare the values between fuzzy coverage probabilities and the expected confidence coefficient by the test of hypothesis. In this example, let *m* = 10,000 and the criterion used in comparing the coverage probability at significance level 95% will be

(33) $$\begin{array}{c} \hat{C}P\geq 0.95-1.645\sqrt{\frac{0.95\left(1-0.95\right)}{10,000}}=0.9464. \end{array}$$

Considering a fuzzy confidence interval at *α*-cut which gives the coverage probability higher than 0.9464 at significant level 95% will be the coverage probability that is covered in the expected confidence coefficient. Then only the coverage probability that is covered in the expected confidence coefficient will be used in the most appropriate expected length estimation. In addition, the fuzzy confidence interval of the *α* − cut which gives the narrowest expected length is considered as the most appropriate expected length. Both lower and upper fuzzy coverage probabilities and expected lengths results are shown in Tables 3 and 4, respectively.

## 4. Numerical Results 

From the numerical example, the fuzzy reliability for the RMSS is calculated. Estimation of fuzzy confidence interval for fuzzy system reliability model $\left({\tilde{\mu }}_{R}\right)$ in each *α* ∈ [0,1] revealed that the fuzzy confidence interval can be divided into 2 parts which are lower fuzzy confidence interval and upper fuzzy confidence interval as shown in Figure 6.

From Figure 6, the lower bound and upper bound of the fuzzy parameter are estimated at a 95% significance level. Next, the performance of the estimated parameter of the fuzzy confidence interval of fuzzy system reliability model is considered. The coverage probability and expected length is used in calculation with the above method with repeated time at *m* = 10,000.

**Figure 7:**
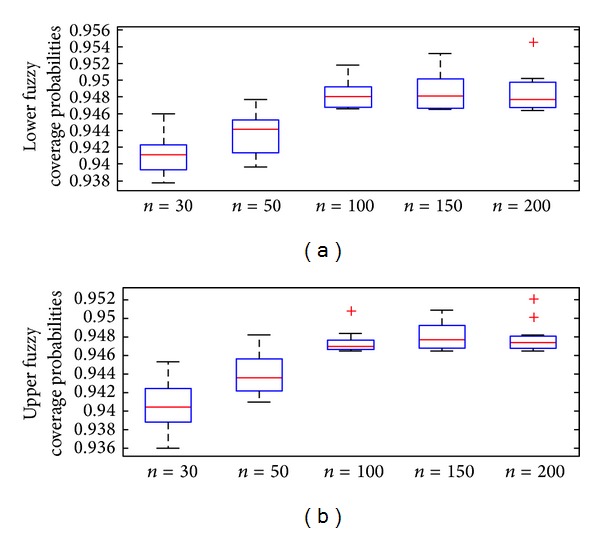
Boxplots of the lower and upper fuzzy coverage probability with sample size *n* = 30,  50, 100, 150, and 200 at significance level 95%.

In Figure 7(a), it is seen that at the sample size *n* = 30,50  (*n* < 100) there are some *α*-cut that give lower coverage probability covering in the expected confidence coefficient when $\hat{C}P\geq 0.9464$. However, if considering, at sample size *n* = 100,150,200  (*n* ≥ 100), the lower coverage probability of every *α* ∈ [0,1] is covering in the expected confidence coefficient, likewise, Figure 7(b) shows that at the sample size *n* = 30,50  (*n* < 100) there are only few *α*-cut numbers that contain upper coverage probability in the expected confidence coefficient at $\hat{\text{C}}P\geq 0.9464$, but, at the sample size = 100,150,200  (*n* ≥ 100), the upper coverage probability of every*α* ∈ [0,1] is in the expected confidence coefficient.

Then only the coverage probability that is covered in the expected confidence coefficient will be used in estimation of the most appropriate expected length. The result shows that the fuzzy confidence interval at *α*-cut = 0.9 gives the narrowest lower expected length. It is considered as the most appropriate lower expected length. It is also seen that the fuzzy confidence interval at *α*-cut = 0.7 gives the narrowest upper expected length. It is considered as the most appropriate upper expected length as shown in Tables 3 and 4.

It seems that at the larger sample size (*n*) more *α*-cut numbers are obtained for the estimated parameter of the fuzzy confidence interval of fuzzy system reliability model. The good interval estimator for the fuzzy confidence interval is the obtained coverage probabilities that can cover the expected confidence coefficient $\hat{C}P\geq 0.9464$ with the narrowest expected length.

## 5. Conclusions and Discussions 

This paper presents an innovative modeling approach when dealing with uncertainties in the RMSS. The Markov process for the RMSS with a fuzzy failure rate and a fuzzy repair rate is considered and the fuzzy system reliability is constructed. Interval estimation of the fuzzy system reliability model is constructed by extending the concepts of two-sided 100(1 − *γ*)% confidence interval of the true parameters to the case where both parameter and random variables are fuzzy based on the central limit theorem. Recently, much research proposed an approach based on fuzzy data for constructing a fuzzy confidence interval, but without considering the performance analysis of interval estimator. In this research, the performance of fuzzy confidence interval is assessed based on the coverage probability and the expected length.

From the study, it is seen that estimation of the fuzzy confidence interval of fuzzy system reliability for RMSS will be effective when the sample size is *n* ≥ 100. This results in lower coverage probability and upper coverage probability which covered in the expected confidence coefficient at *α* ∈ [0,1] and the narrowest lower expected length when *α*-cut = 0.9 and the narrowest upper expected length when *α*-cut = 0.7. Accordingly, we conclude that the model presented herein is an effective estimation method at *α*-cut = 0.9 and *α*-cut = 0.7. This study is suitable for the system reliability of multistate system where the accurate data are fuzzy values. In further work, fuzzy confidence intervals of system reliability for more complex systems are created. In particular, performance of interval estimator is also being considered based on the coverage probability and the expected length.

## Figures and Tables

**Table 1:** Triangular fuzzy number of fuzzy failure rates and fuzzy repair rates (per year).

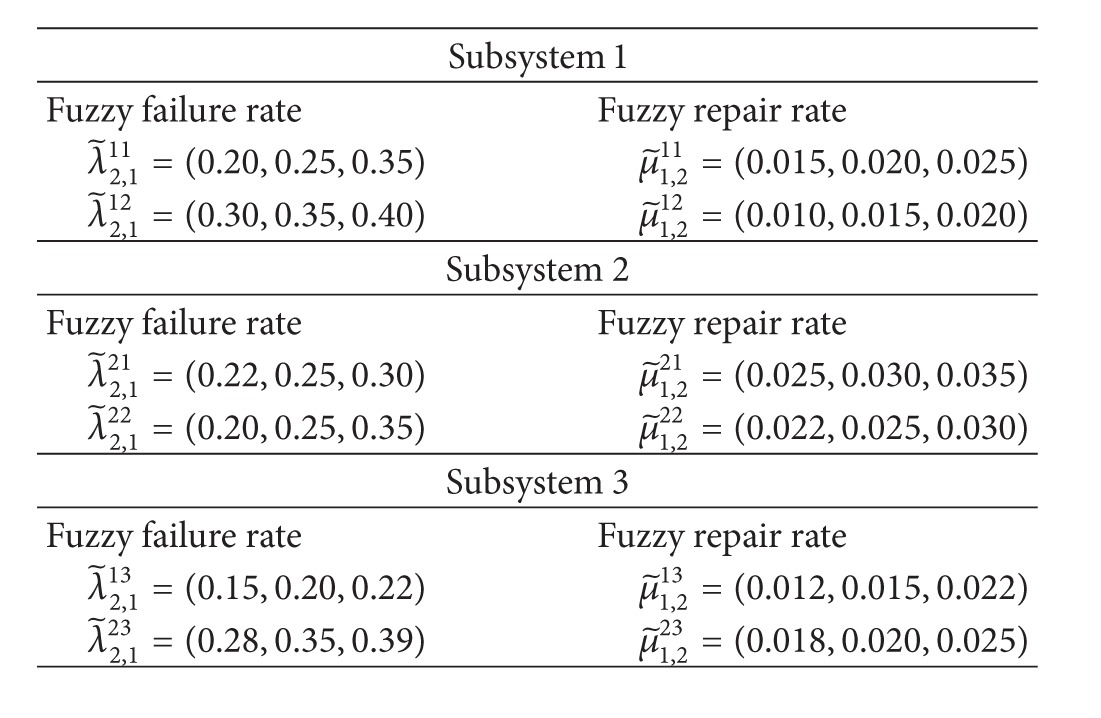

**Table 2:** The fuzzy parameter of fuzzy system reliability for RMSS in each *α* ∈ [0,1].

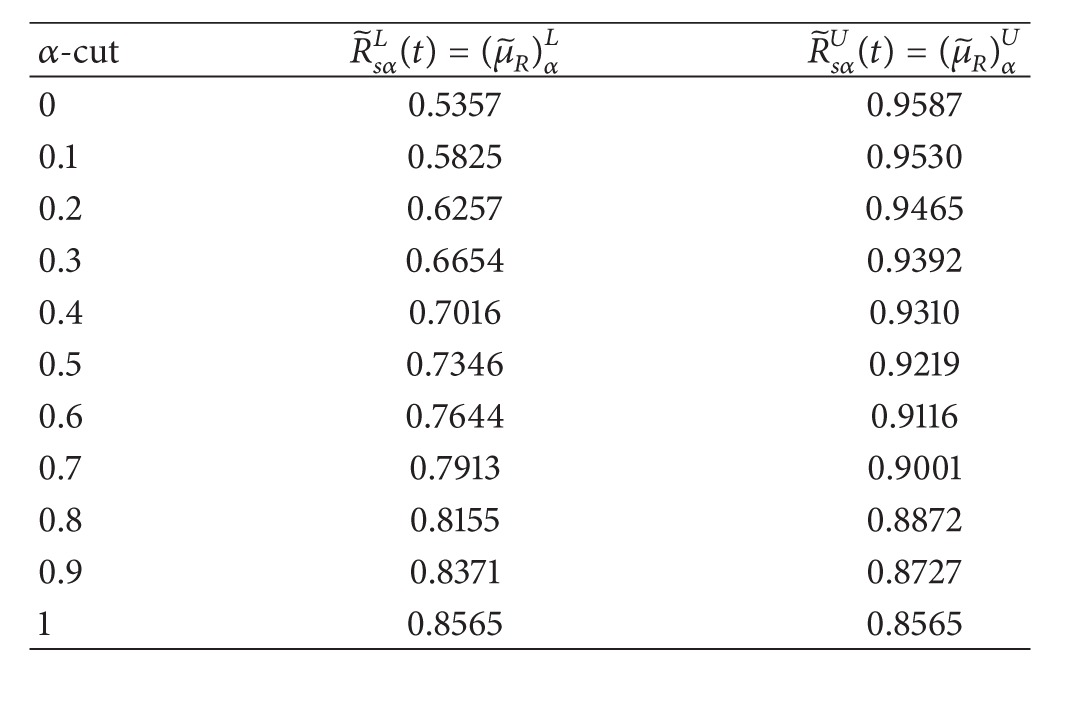

**Table 3:** Lower fuzzy coverage probabilities and lower expected lengths for 95% confidence interval where *m* = 10,000 and $\hat{C}P\geq 0.9464$.

*α*-cut	*n* = 30		*n* = 50		*n* = 100		*n* = 150		*n* = 200	
	Lower *CP*	Lower *EL*	Lower *CP*	Lower *EL*	Lower *CP*	Lower *EL*	Lower *CP*	Lower *EL*	Lower *CP*	Lower *EL*
0	0.9402	0.7091	0.9456	0.5524	**0.9485**	0.3908	**0.9532**	0.3196	**0.9500**	0.2769
0.1	0.9377	0.7102	0.9426	0.5518	**0.9472**	0.3908	**0.9494**	0.3199	**0.9464**	0.2769
0.2	0.9390	0.7089*	0.9440	0.5516	**0.9467**	0.3909	**0.9466**	0.3196	**0.9487**	0.2768
0.3	0.9425	0.7090	0.9443	0.5518	**0.9489**	0.3908	**0.9465**	0.3196	**0.9467**	0.2768
0.4	0.9443	0.7100	0.9409	0.5521	**0.9518**	0.3914	**0.9467**	0.3201	**0.9491**	0.2772
0.5	0.9414	0.7093	0.9441	0.5513*	**0.9493**	0.3908	**0.9465**	0.3195	**0.9468**	0.2767
0.6	0.9411	0.7088	0.9409	0.5516	**0.9466**	0.3913	**0.9504**	0.3196	**0.9502**	0.2769
0.7	0.9387	0.7101	**0.9465**	0.5520	**0.9480**	0.3911	**0.9476**	0.3195	**0.9467**	0.2769
0.8	0.9460	0.7109	0.9396	0.5515	**0.9469**	0.3911	**0.9482**	0.3194	**0.9477**	0.2770
0.9	0.9417	0.7090	**0.9477**	0.5513*	**0.9466**	0.3907*	**0.9481**	0.3193*	**0.9475**	0.2766*
1	0.9408	0.7102	0.9442	0.5515	**0.9510**	0.3908	**0.9511**	0.3198	**0.9555**	0.2769

Coverage probabilities that are higher than the expected confidence coefficient are shown in bold numbers.

*The narrowest expected length.

**Table 4:** Upper fuzzy coverage probabilities and upper expected lengths for 95% confidence interval where *m* = 10,000 and $\hat{C}P\geq 0.9464$.

*α*-cut	*n* = 30		*n* = 50		*n* = 100		*n* = 150		*n* = 200	
	Upper *CP*	Upper *EL*	Upper *CP*	Upper *EL*	Upper *CP*	Upper *EL*	Upper *CP*	Upper *EL*	Upper *CP*	Upper *EL*
0	0.9392	0.7092	0.9460	0.5514	**0.9469**	0.3908	**0.9465**	0.3197	**0.9501**	0.2769
0.1	0.9435	0.7112	**0.9466**	0.5511	**0.9466**	0.3910	**0.9494**	0.3194	**0.9482**	0.2768
0.2	0.9404	0.7117	**0.9482**	0.5516	**0.9475**	0.3909	**0.9483**	0.3196	**0.9465**	0.2768
0.3	0.9360	0.7098	0.9425	0.5516	**0.9470**	0.3914	**0.9477**	0.3197	**0.9470**	0.2771
0.4	0.9384	0.7092	0.9441	0.5506*	**0.9484**	0.3911	**0.9475**	0.3193	**0.9470**	0.2767
0.5	0.9411	0.7101	0.9444	0.5514	**0.9465**	0.3912	**0.9474**	0.3197	**0.9477**	0.2769
0.6	0.9420	0.7101	0.9410	0.5517	**0.9509**	0.3908	**0.9465**	0.3196	**0.9467**	0.2768
0.7	0.9426	0.7084	0.9421	0.5511	**0.9477**	0.3906*	**0.9466**	0.3191*	**0.9474**	0.2765*
0.8	0.9400	0.7096	0.9425	0.5514	**0.9475**	0.3910	**0.9508**	0.3195	**0.9466**	0.2768
0.9	0.9387	0.7075*	0.9436	0.5522	**0.9469**	0.3907	**0.9509**	0.3198	**0.9475**	0.2768
1	0.9453	0.7103	0.9412	0.5509	**0.9466**	0.3907	**0.9489**	0.3195	**0.9525**	0.2767

Coverage probabilities that are higher than the expected confidence coefficient are shown in bold numbers.

*The narrowest expected length.
